# Antiproliferative effect of dexamethasone in the MCF-7 breast cancer cell line

**DOI:** 10.3892/mmr.2015.3920

**Published:** 2015-06-12

**Authors:** FREDERIC BUXANT, NADÈGE KINDT, GUY LAURENT, JEAN-CHRISTOPHE NOËL, SVEN SAUSSEZ

**Affiliations:** 1 Department of Gynecology, Iris South Hospital, 1050 Brussels, Belgium; 2 Laboratories of Anatomy and Cell Biology, University of Mons, 7000 Mons, Belgium; 3 Histology, Faculty of Medicine and Pharmacy, University of Mons, 7000 Mons, Belgium; 4 Department of Pathology, Erasme Hospital, Free University of Brussels, 1070 Brussels, Belgium

**Keywords:** glucocorticoid receptor, MCF-7

## Abstract

Glucocorticoids (GCs) are used in the treatment of cancer to induce programmed cell death in the transformed cells of the hematopoietic system and to reduce side effects. Additionally, GCs are described as an inhibitor of certain chemotherapy or radiation-induced apoptosis and also an inhibitor of cancer progression by downregulating or upregulating the expression of several genes. The present study used immunofluorescence to investigate the presence of the glucocorticoid receptor (GR) in MCF-7 cells, and the cell culture growth was determined by cell counting the number of cells following exposure to GC and/or dexamethasone (Dex). The presence and immunoreactivity of the GR were confirmed, and treatment with Dex (10^−8^–10^−7^ M) caused an inhibitory effect (30–35%) on the proliferative activity of the MCF-7 cells. This growth inhibitory effect was possibly produced by the pro-apopotic effect of Dex. Since Dex is administered systematically prior to breast cancer chemotherapy, the possible interactions between these drugs require further investigation.

## Introduction

Glucocorticoids (GCs) are essential steroid hormones in mammals, which are important as modulators of the inflammatory response and are involved in a multitude of cellular processes, including cell differentiation and metabolism, the immune response and cell apoptosis (1). GCs are used therapeutically for the treatment of inflammatory and autoimmune diseases, allergic reactions and soft tissue edema following solid organ transplantation, and also in the therapeutic induction of apoptotic cell death in malignant lymphoid cells (2). Prior to, during and subsequent to chemotherapy for the treatment of solid tumors, GCs, usually dexamethasone (Dex), are administered at various doses to reduce toxicity, particularly hyperemesis, and to protect the normal tissues of the patients against the long-term effects of genotoxic drugs (3). In terms of breast cancer, taxanes, including paclitaxel, are among the most active chemotherapeutic agents used (4). Initially, taxane use was limited due to hypersensitivity reactions, however, following improvements in management, largely by pre-treatment with GC, taxane chemotherapy has become part of standard treatment in the majority of western countries (4).

However, data from preclinical and clinical investigations suggest that GCs may induce tumor resistance to apoptosis and, therefore, cause less sensitivity to chemotherapy in various types of solid neoplasm, including osteosarcoma, brain tumor, breast and cervical carcinoma, melanoma and neuroblastoma (5,6).

As with other steroid hormones, GCs exert their effects on target cells via binding to cognate nuclear glucocorticoid receptors (GRs), which function as ligand-regulated transcription factors (7). Ligand-bound GRs induce gene transactivation either directly, by interacting with glucocorticoid response elements (GREs) that are located in regulatory sequences of target genes, or indirectly, by interacting with other transcription factors, including activator protein-1, specificity protein 1 or nuclear factor-κB. By doing so, GCs can positively or negatively regulate the expression of a wide array of target genes (7).

Cancer is diagnosed in >1,000,000 females annually in the European community, ~30% of which are breast cancer (8). The majority (80%) of these cases of breast cancer are hormone sensitive, and existing systemic treatments can be targeted (herceptin hormone therapy) or non-targeted (chemotherapy) (4). The systemic administration of GCs does not appear to affect the risk of breast cancer (9). *In vitro*, GCs antagonize estrogen actions via the activation of the estrogen sulfotransferase (10). Skor *et al* (11) suggested that pretreatment with mifepristone offered a useful strategy for increasing tumor cell apoptosis in chemotherapy-resistant GR^+^ triple negative breast carcinoma.

Although the action of GCs on breast cancer cells remain to be fully elucidated, they are frequently prescribed and systematically combined with the prescription of the majority of chemotherapeutic agents (5).

It is, therefore, essential to evaluate the direct role of GCs on cancer cells. The present study aimed to investigate the presence and reactivity of GRs, and to examine the effect of applying the Dex GC on an MCF-7 breast cancer cell line.

## Materials and methods

### Cell line and culture

The MCF-7 cells (obtained from Professor G. Leclercq, J.-C. Heuson Breast Cancer Translational Research Laboratory, Institute Jules Bordet, Free University of Brussels, Brussels, Belgium) were maintained at 37°C in a cell incubator, with a humid atmosphere of 5% CO_2_. Unless specified otherwise, the cells were cultured in T-flasks, containing Dulbecco's modified Eagle's medium (DMEM), supplemented with Phenol Red, 10% fetal bovine serum (FBS; Hyclone, Logan, UT, USA), 25 mM N-2-hydrox yethylpiperazine-N′-2-ethanesulfonic acid, 2 mM L-glutamine and 1X antibiotic/antimycotic mix (all from Lonza, Verviers, Belgium). For the investigation of nuclear receptors by immunofluorescence microscopy, the cells were seeded in Phenol Red-free DMEM, supplemented with 10% charcoal-stripped FBS (EFM; Hyclone).

### Measurement of cell culture growth by cell counting

The MCF-7 cells were plated at a density of 10^4^ cells/cm^2^ in 12-well plates at 37°C. The following day, the media of the cell cultures were replaced with fresh medium, with or without Dex (Sigma-Aldrich, St. Louis, MO, USA) (10^−7^, 10^−8^ and 10^−9^ M). The measurement of cell culture density was performed 3 days after treatment. The cells were dislodged from the vessel bottom by treatment with 1 ml trypsin-EDTA solution (Lonza). Following vigorous pipetting, the concentrations of the cells in the suspension were determined using an electronic cell counter (Z1 Coulter counter; Beckman Coulter, Fullerton CA, USA).

### Immunofluorescence microscopy

The MCF-7 cells were plated in EFM, at a density of 5,000 cells/cm^2^, on sterile round glass coverslips in 12-well dishes at 37°C. Following 3 days of growth, the cells were treated with 10^−7^ Dex for 30 min or 6 h. At the end of the hormone exposure, the cell monolayers were fixed for 20 min with 4% paraformaldehyde (Sigma-Aldrich) in Dulbecco's phosphate-buffered saline (DPBS; Sigma-Aldrich). Following fixation, the paraformaldehyde was replaced with DPBS, and the cell cultures were stored at 4°C until immunostaining. Prior to the application of antibodies, the cell monolayers were rinsed three times with PBS (5 min/wash), containing 0.04 M Na_2_HPO_4_, 0.01 M KH_2_PO_4_, 0.12 M NaCl (pH 7.2) and 0.1% Triton X-100 (Sigma-Aldrich). The same detergent-containing buffer was used for subsequent incubations and rinsing steps. The cells were pre-incubated for 20 min in PBS, containing 0.05% casein (Sigma-Aldrich), to prevent the non-specific adsorption of immunoglobulins (Igs). The cells were then exposed to the primary antibody (mouse monoclonal anti-GR antibody 4H_2_; cat. no. 34–473; Novocastra Laboratories, Ltd., Newcastle upon Tyne, UK) diluted 1:20 in PBS, containing 0.05% casein, for 60 min at room temperature. This was followed by 30 min of exposure to peroxidase-conjugated anti-mouse Ig (ImmPRESS; cat. no. MP-7402; Vector Laboratories, Inc., Burlingame, CA, USA). The cells were subsequently incubated for 30 min at room temperature in the presence of rabbit anti-peroxidase antibody (1:200). Following 30 min incubation, the cell cultures were exposed for 30 min to biotinylated goat anti-rabbit IgG (1:50; cat. no. BA-1000; Vector Laboratories, Inc.). Labeling was completed by exposing the cells for a further 30 min to Texas Red conjugate streptavidin (1:50; Vector Laboratories, Inc.) at room temperature. Following three final rinses in PBS, the coverslips were mounted onto glass slides using commercial anti-fading medium (Vectashield; Vector Laboratories).

The cell preparations were examined using a Leitz Orthoplan microscope equipped with a Ploem system (Leica Microsystems Belgium BVBA, Diegem, Belgium) for epi-illumination. An excitation wavelength of 560 nm and an emission wavelength of 590 nm were used for the observation of Texas Red fluorescence. Images of the cells were captured using a PC-driven digital camera (Leica DC 300F; Leica Microsystems AG, Heerbrugg, Switzerland) operated with Leica IM50 1.2 software. Quantitative analysis of the fluorescence signals was performed on digitalized images using Image J 1.45s software (National. Institutes of Health, Bethesda, MD, USA). The images were analyzed in the red channel following RGB split. The Gray levels, corresponding to the fluorescence intensity, were determined on a scale between 0 and 225 in 50 control cells and 51 treated cells. Distribution histograms were plotted using SigmaPlot^®^ 11 software (Systat Software, Inc., Hounslow, UK).

### Statistical analysis

SigmaPlot^®^ 11 (Systat Software, Inc., Hounslow, UK) software was used for statistical analyses. Parametric analysis was performed using analysis of variance for more than two groups and pairwise comparisons were performed using the Holm-Sidak method or Student's t-test. P<0.05 was considered to indicate a statistically significant difference.

## Results

### MCF-7 cells express GR

**Figure 1:**
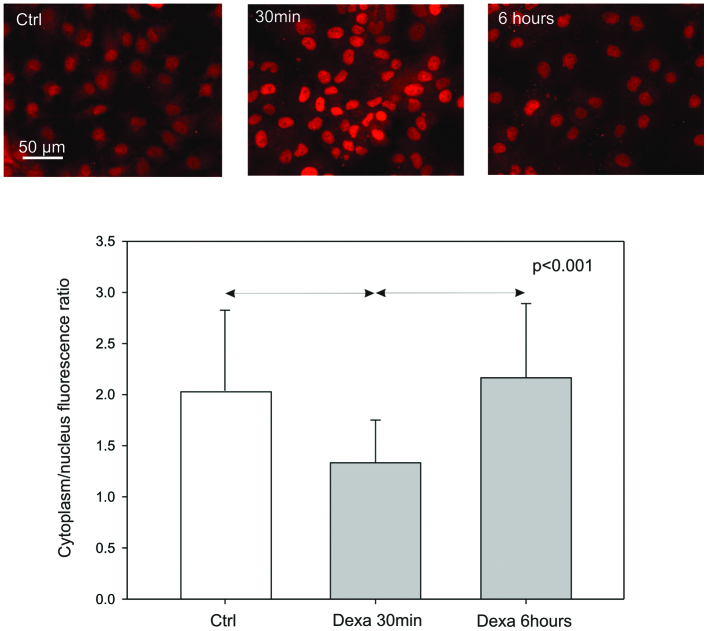
Intensity of the fluorescence of the glucocorticoid receptor in MCF-7 cells treated with 10^−7^ M Dexa for 30 min or 6 h. A marked increase in the nuclear immunofluorescence signal was observed after 30 min exposure. Treatment for 6 h with Dexa resulted in a decrease in nuclear fluorescence. Redistribution of fluorescence during treatment determined by the cytoplasm/nucleus ratio (P<0.001, analysis of variance). Ctrl, control; Dexa, dexamethasone.

The expression of GR in the MCF-7 cells was observed by immunofluorescence microscopy, as shown in Fig. 1. In the absence of steroid hormones, the immunoreactive GR exhibited an ill-defined distribution, which appeared in the cytoplasm and the nuclei. A marked change was observed following 30 min exposure to 10^−7^ M Dex, with a marked increase in the immunofluorescence signal in the cell nuclei (Fig. 1; 30 min). Treatment of the cells for 6 h with Dex resulted in a decrease in nuclear fluorescence. Changes in the immunofluorescence pattern were quantified and expressed as the cytoplasm/nucleus ratios. As shown in Fig. 1, treatment with Dex for 30 min produced a significant decrease in the fluorescence signal in the cytoplasm relative to the that of the nucleus.

### Dex inhibits the proliferation of MCF-7 cells

**Figure 2:**
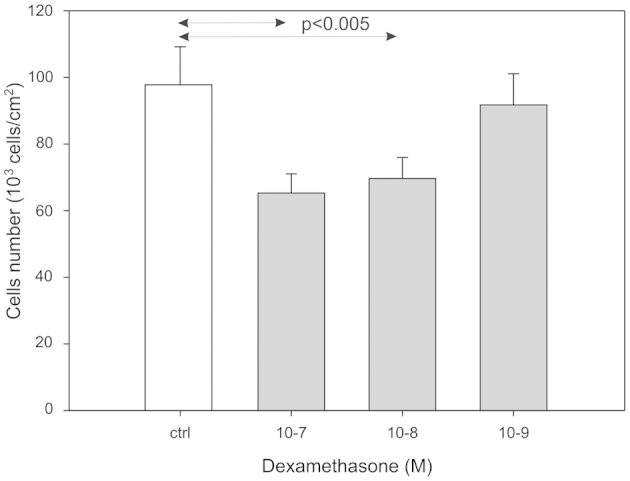
Effect of dexamethasone on the proliferation of MCF-7 cells. Treatment with 10^−7^ and 10^−8^ M dexamethasone significantly decreased the cell culture growth (P<0.005, t-test).

The impact of Dex on MCF-7 cell proliferation was assessed by cell counting following 3 days of culture in the presence of increasing concentrations of Dex. As shown in Fig. 2, higher concentrations of Dex (10^−8^–10^−7^ M) exerted an inhibitory effect (30–35%) on the proliferative activity of the MCF-7 cells.

## Discussion

The expression of GRs by different cancer cells, including breast cancer cells, has been previously observed (12–15). In the present study, immunochemistry revealed the presence of GR in MCF-7 cells. In our previous study, it was observed that a loss of GR appears in poorly differentiated breast cancer cells (13). As with other previous studies (16,17), the first aspect of the present study demonstrated the presence of GR in MCF-7 cells. This receptor was intracellular and present in the cytoplasm and the nucleus.

Exposure of the MCF-7 cells to Dex for 30 min (Fig. 1) was associated with a significant migration (P<0.001) of the receptor to the nucleus. This demonstrated the reactivity of the receptor. Notably, prior to the binding of the GCs, the receptor is in an inactive state in the cytoplasm, associated with proteins, including heat shock protein (HSP)-90, HSP-70 and HSP-56 immunophilins. When GCs bind to the C-terminus, they cause the activation of the receptor and the release of HSP and immunophilins. The GC/GR complex then passes the nuclear membrane to localize in the nucleus and to associate with specific DNA sequences, termed GRE, located in the promoter regions of certain genes (18,19). Following a longer exposure to GC (6 h), the GR was predominantly localized in the cytoplasm. A negative feed back loop is known to exist for nuclear receptors following prolonged exposure to the ligand (18), which may explain the result observed.

Following exposure to Dex at a concentration of 10^−7^ or 10^−8^ M, a significant inhibition in cell proliferation was demonstrated. These results were similar to those of Lippman *et al* (20), in which a reduction in proliferation was observed in up to 50% of MCF-7 cultures. This inhibition is mediated by the inhibition of insulin growth factor-induced cell growth (21). The anti-angiogenic effect of Dex cannot be incriminated in the MCF-7 culture model and, in this cell line, the antiproliferative effect of GCs, therefore, predominates over the anti-apoptotic effect (22). However, the anti-apoptotic effect has been investigated and is mediated by the induction of the expression of genes, which are frequently associated with protection against cell apoptosis, including B-cell lymphoma (Bcl) extra-large, Bcl-2 homologous antagonist killer, serum and glucocorticoid-regulated kinase 1 and mitogen-activated protein kinase phosphatase-1 (22–24). Concomitantly, Dex also reinforces its survival effect by downregulating pro-apoptotic genes (23).

This antiproliferative effect of Dex on cell types, including MCF-7, estrogen receptor^+^ and progesterone receptor^+^, is reassuring, however, it does not guarantee the safe use of Dex in patients with hormone-dependent breast neoplasia. Although a chemosensitization effect has been observed following pre-treatment with Dex on MCF-7 cells exposed to carboplatin (25), such results require confirmation in currently used chemotherapeutic agents, including taxanes. Therefore, chemoresistance has been observed in previous studies and is dependent only on the dosing regimen of Dex (25).

Further investigations are required using chemotherapy and current GC regimens. Similarly, although GCs are not often administered concomitantly with hormone therapies, including tamoxifen and aromatase inhibitor, their potential interaction requires investigation. It is understood that GCs decrease free-estrogens (10), however, whether the GCs exhibit potentiating or inhibitory effects on cells occurs following exposure to such drugs remains to be elucidated. Finally, the identification of GR in virtually non- or entirely non-hormone-dependent breast cancer cells, including triple negative breast carcinoma) (11), encourage further investigation into the action of GC in all types of breast cancer.

In conclusion, the action of GCs has been investigated in several cell lines, either alone or in the presence of chemotherapeutic agents. The present study demonstrated the antiproliferative action of Dex on MCF-7 cells.

As GC is used during all chemotherapeutic treatments, this effect requires confirmation in the presence of these agents and adhering to the administration regimens of different molecules.
